# Simulation Training for Falls on the Older Adult Mental Health Wards

**DOI:** 10.1192/bjo.2025.10316

**Published:** 2025-06-20

**Authors:** Naomi Tomlinson, Emma Baxey, Sam Ter-Host, Anita Bignall, Hannah Iannelli

**Affiliations:** Maudsley Learning, London, United Kingdom

## Abstract

**Aims:** Falls are the most commonly reported patient safety incident in all older adult (OA) inpatient wards, and studies suggest there may be up to three times as many falls in OA mental health, compared with physical health settings. Many factors present on the mental health wards may influence this, including higher levels of agitation, psychotropic side-effects, a culture of promoting recovery through activity and a higher prevalence of side rooms resulting in less direct observation. There are four OA mental health wards within South London and the Maudsley NHS Trust. Following an analysis of serious incidents, falls prevention and management was identified as an area for improvement. Work to date has included updating the clinical falls policy, promoting a ‘falls awareness week’ and introducing a mandatory falls e-learning module. However, concerns remained about the practical application of this learning. As such we developed and delivered a half-day simulation course, with the aim of engaging staff in a enjoyable, practical session which would allow for reflective discussions and embed the new falls policy within ward culture.

**Methods:** The simulation course is designed to reach approximately 100 multi-professional staff across the four wards. Eight deliveries have taken place, or are scheduled to take place, between December 2024 and March 2025. Learning objectives, which were informed by trust protocols and incident reports, included increasing confidence with: identifying physical signs and symptoms of fractures and head injuries; initial assessments, management, ongoing review, and escalation process following a fall; the role of team working, handover and communication with family and colleagues; documentation and reporting systems after falls incidence, and increased awareness of the tools available to assist on the intranet.

Each delivery is co-facilitated by simulation faculty and ward staff. The course features four simulated patients, portrayed by actors. The scenarios are designed to each include different risk factors, mechanisms and consequences of falls. Each scenario is followed by a reflective modified diamond debrief.

**Results:** Pre and post-course questionnaires currently show increased confidence with regard to all the learning objectives. A thematic analysis of free text comments will also be presented, alongside reflections from the facilitators.

**Conclusion:** Simulation using live actors is an under-utilised medium for training in situations where physical and mental health presentations co-occur, and can be instrumental in embedding new policies or learning from serious incidents.

